# Plasma biomarkers and their correlation in adult children of parents with Alzheimer’s disease

**DOI:** 10.3389/fnagi.2022.977515

**Published:** 2022-08-30

**Authors:** Ling-Chun Huang, Ming-Hui Chen, Chih-Pin Chuu, Kuan-Ying Li, Tzyh-Chyuan Hour, Yuan-Han Yang

**Affiliations:** ^1^Department of Neurology, Kaohsiung Municipal Ta-Tung Hospital, Kaohsiung Medical University, Kaohsiung City, Taiwan; ^2^Department of Neurology, Kaohsiung Medical University Hospital, Kaohsiung City, Taiwan; ^3^Neuroscience Research Center, Kaohsiung Medical University, Kaohsiung City, Taiwan; ^4^Institute of Cellular and System Medicine, National Health Research Institutes, Zhunan, Miaoli County, Taiwan; ^5^Department of Biochemistry, College of Medicine, Kaohsiung Medical University, Kaohsiung City, Taiwan; ^6^Post-baccalaureate Medicine, Kaohsiung Medical University, Kaohsiung City, Taiwan

**Keywords:** Alzheimer’s disease, amyloid–beta, tau, plasma biomarker, family history

## Abstract

Family history (FH) of late-onset Alzheimer’s disease (AD) is associated with changes in several cerebrospinal fluid (CSF) biomarkers in cognitively normal individuals. However, potential changes in plasma biomarkers remain unknown. This study aimed to evaluate potential plasma biomarkers and their correlation in cognitively normal adult children (AC) and to compare this data with their AD parents and unrelated non-demented controls (NC). Participants with dementia due to AD, their AC and NC were recruited. Plasma samples were assessed for amyloid beta (Aβ)_1–42_, Aβ_1–40_, total tau (T-tau) and phosphorylated tau (P-tau). Kruskal–Wallis test was used for the comparison of this data between the three groups. Spearman rank correlation was used for evaluation of the correlations between Aβ_1–40_ and Aβ_1–42_, and T-tau and P-tau in the AD and AC groups. A total of 99 subjects completed the assessment (30 had AD; 38 were AC group; and 31 were NC). Compared with the NC group, there were significantly higher levels of Aβ_1–40_, P-tau, and P-tau/T-tau ratio, and lower levels of Aβ_1–42_ and Aβ_1–42_/Aβ_1–40_ ratio in the AD and AC groups. The correlation between the level of Aβ_1–42_ and Aβ_1–40_ and level of T-tau and P-tau was only observed in the AC but not in the AD group. AC of AD parents demonstrate some indicators of AD like their parents. Disruption to the correlation between Aβ and tau in AD may be a biomarker for the development of AD in AC, which should be examined in a longitudinal cohort.

## Introduction

Recent advances suggest that Alzheimer’s disease (AD) has a lengthy period in which cerebral lesions gradually accumulate in the absence of clinical symptoms, eventually causing sufficient synaptic and neuronal damage that results in symptomatic AD (Price and Morris, 1999; Price et al., 2009; Jack et al., 2010). Potential disease-modifying therapies for AD may be most beneficial when initiated in the preclinical stage, before the occurrence of irreversible brain damage (Dubois et al., 2016). Therefore, it is crucial to increase the capacity to identify individuals at high risk for developing symptomatic AD not only for research purpose but in clinical practice. “Antecedent Biomarkers of AD: The Adult Children Study (ACS)” is a longitudinal study which has been conducted by Washington University since 2005; it aims to validate biological markers, imaging features, and other indicators of preclinical AD (Coats and Morris, 2005). This study revealed that family history (FH) for AD is associated with age-related changes in several cerebrospinal fluid (CSF) biomarkers in cognitively normal individuals, independent of the ε4 allele of apolipoprotein E (APOE ε4) (Xiong et al., 2011).

However, given the invasiveness of the CSF collection method, these biomarkers are not used extensively in clinical settings. Plasma is a more convenient and applicable biomarker, and increasing evidence has demonstrated the applicability of plasma biomarkers. Studies regarding the genetic risk of AD in Down syndrome showed higher concentrations of plasma amyloid beta (Aβ)_1–42_ in individuals with Down syndrome compared with the controls (Mengel et al., 2020; Hendrix et al., 2021). Higher levels of Aβ_1–42_ and a higher Aβ_1–42_/Aβ_1–40_ ratio were noted in mutation carriers in the preclinical and clinical stages of autosomal-dominant AD (Quiroz et al., 2015). Plasma measurements of Aβ and tau are increasingly showing a positive predictive value for AD-related neuropathology in patients with mild cognitive impairment and AD (Risacher et al., 2019; Li et al., 2022). Meanwhile, plasma amyloid and tau levels may predict cognitive decline and subsequent AD dementia (van Oijen et al., 2006; Graff-Radford et al., 2007; Abdullah et al., 2009; Chouraki et al., 2015; Janelidze et al., 2020; Nam et al., 2020; Cullen et al., 2021).

Family history for AD as a risk factor for developing AD and cognitive decline has been well documented (Huang et al., 2004; Jayadev et al., 2008), and changes in CSF biomarkers in the adult children (AC) of parents with AD has been previously reported (Xiong et al., 2011). However, studies regarding the potential of plasma biomarkers in these AC are limited and most of them only focus on the level of Aβ compared with normal controls (Ertekin-Taner et al., 2001, 2008). To the best of our knowledge, the plasma Aβ_1–42_/Aβ_1–40_ ratio, total tau (T-tau) and tau phosphorylated at threonine 181 (P-tau 181) in this population and a comparison of these potential biomarkers between AC and their AD parents has not been previously addressed.

The Aβ_1–42_/Aβ_1–40_ ratio is more strongly associated with tau and clinical progression than Aβ_1–42_ or Aβ_1–40_ alone (Risacher et al., 2019; Delaby et al., 2022). Therefore, we conducted the current study to evaluate the plasma biomarkers (Aβ_1–42_, Aβ_1–40_, Aβ_1–42_/Aβ_1–40_ ratio, T-tau and P-tau 181) and their correlations in cognitively normal individuals with AD parents and compared this data with their parents and non-demented controls (NC) (unrelated controls). The hypothesis is that the AC of AD parents who potentially have AD, will demonstrate indicators of AD at a greater frequency than the normal controls and this will demonstrate that these plasma indicators are antecedent biomarkers of AD.

## Materials and methods

### Recruitment of participants

From March 2020 patients with dementia due to AD, their AC and unrelated normal controls were recruited from the neurological outpatient department of Kaohsiung Municipal Ta-Tung Hospital, Southern Taiwan. We modified the Washington University ACS study (Coats and Morris, 2005) for use in Taiwan. The average age of dementia diagnosis in Taiwan is 79, while their children’s age is around 55 (Wang et al., 2014). Participants aged 50–74 years old who had at least one biological parent with probable AD followed up regularly at our outpatient department were recruited as the AC group. Participants with depression or those who already had dementia were excluded. Stable AD patients who had been receiving acetylcholinesterase inhibitors for at least 12 months were recruited as the AD group. The diagnosis of AD was based on the National Institute of Neurological and Communicative Disorders and Stroke-Alzheimer’s Disease and Related Disorders Association (NINCDS-ADRDA) criteria (McKhann et al., 1984). Cognitively normal individuals without dementia, as determined by a Clinical Dementia Rating^®^ (CDR^®^) (Morris, 1993) score of zero and an instrument of ascertainment of dementia 8 (AD8) (Galvin et al., 2005) score of <2 conducted by an experienced physician and who did not have a FH of AD, were recruited as the NC group. The NC individuals recruited in our study were volunteers selected from outpatients at the neurology clinic. All the subjects enrolled were not carrying any pathological mutation responsible for AD or familial AD. The participants and their relatives were informed of the details of the study. The Kaohsiung Medical University Hospital Institutional Review Board [KMUHIRB-SV(I)-20190025 and KMUHIRB-G(I)-20210026] approved the study protocol and the participants provided written informed consent prior to their inclusion.

### Cardiovascular risk factors

At baseline visits, a self-administered structured questionnaire was provided to collect sociodemographic information, medical history, and medication exposure. Total cholesterol and glucose levels were measured from venous blood after fasting for at least 8 h. A calibrated standard aneroid sphygmomanometer was used to measure blood pressure after resting for 5 min. Two measurements of blood pressure were taken, and the average of these two measurements was used for analysis. Hypertension was defined as systolic or diastolic blood pressure of ≥140/90 mmHg or the use of antihypertensive medications. Diabetes was defined as a fasting blood glucose level of ≥126 mg/dl or current treatment for diabetes. Hypercholesterolemia was defined as a serum total cholesterol level of ≥200 mg/dl or the use of lipid-lowering medications. The Cardiovascular risk factors, aging, and incidence of dementia (CAIDE) dementia risk score was used to estimate the risk of dementia 20 years later, based on age, sex, education, systolic blood pressure, body mass index, total cholesterol, and physical activity (Kivipelto et al., 2006). No collected data were available for physical activity, and this variable was not included in the risk score. We used the version of CAIDE score calculated adding points for the presence of the APOE ε4 allele (Enache et al., 2016). The maximum number of points for the version of CAIDE score was 17. The score was calculated for the participants in the AC and NC groups.

### Clinical and cognitive assessments

A series of neuropsychological assessments were conducted for each recruited participant, including the AD8, Mini-Mental State Examination (MMSE) (Folstein et al., 1975), CDR and Center for Epidemiological Studies Depression Scale (CESD) (Radloff, 1977). These were used to evaluate the participant’s clinical status, depression status, and cognitive function. The neuropsychological assessments were conducted by a senior neuropsychologist and an experienced physician based on information from a knowledgeable collateral source (usually a spouse or adult child).

### DNA preparation

DNA was extracted from 5 ml whole blood which was collected in ethylenediaminetetraacetic acid (EDTA)-anticoagulated blood tubes. Genomic DNA was extracted using the PureLink™ Genomic DNA Mini kit (Invitrogen, Waltham, MS, United State, K1820-02), according to the manufacturer’s guidelines. The isolated genomic DNA samples were stored at −20°C prior to further analysis.

### Apolipoprotein E genotyping

Apolipoprotein E genotyping was conducted for all participants. Genotyping was performed using a TaqMan-based real-time PCR assay (Applied Biosystems^®^ by Life Technologies, N8010560). The APOE gene copy number was determined using Applied Biosystems’ commercially available TaqMan Copy Number Assays, Apolipoprotein C-I (Assay ID: C__3084793_20 and C__904973_10). For the TaqMan Single Nucleotide Polymorphism (SNP) Genotyping Assay, 3 μl of TaqMan Genotyping Master Mix (Applied Biosystems^TM^, Waltham, MS, United States, P/N: 4371355) and 1 μl of genomic DNA (10 ng/μl) were used. After a pre-PCR hold at 50°C for 2 min, 40 reaction cycles were performed using the Applied Biosystems 7,500 Real-Time PCR System (Applied Biosystems^TM^, Waltham, MS, United States) with the following thermal cycling conditions: hold at 95°C for 10 min for initial denaturation and enzyme activation, followed by 40 cycles of 95°C for 15 s and 60°C for 1 min.

### Plasma sample for ELISA

Plasma samples were collected in EDTA vacutainers, which were centrifuged for 15 min at 4,000 rpm. After centrifugation, each sample was stored at −80°C. All samples were incubated at room temperature before being measured. Quantification of Aβ_1–42_, Aβ_1–40_, T-tau, and P-tau 181 in the plasma was performed using specific ELISA kits (Human Amyloid β (1–40) Assay Kit–Invitrogen, code number KHB3481; Human Amyloid β (1–42) Assay Kit–Invitrogen, code number KHB3441; Human Tau (Total) Assay Kit–Invitrogen, code number KHB0041) and Human Tau (pT181) Assay Kit–Invitrogen, code number KHO0631). All assays were performed according to the manufacturer’s instructions. All reagents were prepared at room temperature (20–25°C) approximately 30 min before use.

### Statistical analysis

Data was presented as the mean ± standard deviation (SD), median (interquartile range) or proportion. For comparison between the AD, AC, and NC groups, the chi-squared test was used for categorical data, and the Kruskal–Wallis test were used for continuous data. The Spearman’s rank correlation test was used to evaluate the correlation of plasma Aβ_1–40_, Aβ_1–42_, T-tau, and P-tau in AD, AC and between these two groups. All analyses were performed using SPSS 26.0 (SPSS Inc., Chicago, IL, United States). A two-tailed *P*-value of <0.05 was considered to indicate a statistically significant difference. Data were visualized using Prism 7 (Graphpad).

## Results

### Demographic characteristics of the participants

Table 1 presents the demographic characteristics, cognitive function, and cardiovascular risk factors of the participants. A total of 99 participants completed the assessments and 30 had AD; 38 were classified as being in the AC group; and 31 were in the NC group. Most of the participants were female (80% in the AD group; 92.1% in the AC group; and 64.5% in the NC group). As expected, the APOE ε4 allele was significantly more frequent in the AD and AC groups (46.7% in the AD group; 39.5% in the AC group; and 16.1% in the NC group, *p* < 0.001). The mean age in years was 82.6 ± 6.2, 57.5 ± 6.7, and 74.2 ± 6.0 in the AD, AC, and NC groups, respectively. The mean educational level in years was 5.3 ± 5.3, 13.7 ± 3.7, and 10.5 ± 3.8, respectively. The mean MMSE score was 13.6 ± 7.2, 28.4 ± 1.7, and 24.5 ± 3.7, respectively. More participants in the AD group had hypertension than the other two groups. The CAIDE score was higher in NC group compared to AC group (7.9 ± 2.3 vs. 6.2 ± 2.0, *p* = 0.005).

**TABLE 1:** Demographic and clinical characteristics of the study participants.

Characteristics	AD (*n* = 30)	AC (*n* = 38)	NC (*n* = 31)	*P*-value
Gender, female (%)	24 (80.0)	35 (92.1)	20 (64.5)	0.002
APOE ε4 positive (%)	14 (46.7)	15 (39.5)	5 (16.1)	<0.001
Age (years)	82.6 ± 6.2	57.5 ± 6.7	74.2 ± 6.0	<0.001
Education (years)^†^	5.3 ± 5.3	13.7 ± 3.7	10.5 ± 3.8	<0.001
MMSE^‡^	13.6 ± 7.2	28.4 ± 1.7	24.5 ± 3.7	<0.001
Hypertension (%)	17 (56.7)	7 (18.4)	7 (22.6)	0.002
Diabetes (%)	10 (33.3)	5 (13.2)	4 (12.9)	0.062
Hypercholesterolemia (%)	14 (46.7)	8 (21.1)	13 (41.9)	0.059
CAIDE score	–	6.2 ± 2.0	7.9 ± 2.3	0.005

Data are shown as the mean ± SD for quantitative variables and *n* (%) for qualitative variables. *P*-value for AD, AC, and NC group using analysis of chi-square (gender, APOE ε4 positive, hypertension, diabetes and hypercholesterolemia) or Kruskal-Wallis tests. ^†^One participant missing data (1 AD). ^‡^One participant missing data (1 AC). AD, Alzheimer’s disease; AC, adult children; NC, non-demented control; APOE, apolipoprotein E; MMSE, Mini-Mental State Examination; CAIDE score, cardiovascular risk factors, aging, and incidence of dementia score.

### The level of plasma biomarkers in the Alzheimer’s disease, adult children, and non-demented control groups

**FIGURE 1:**
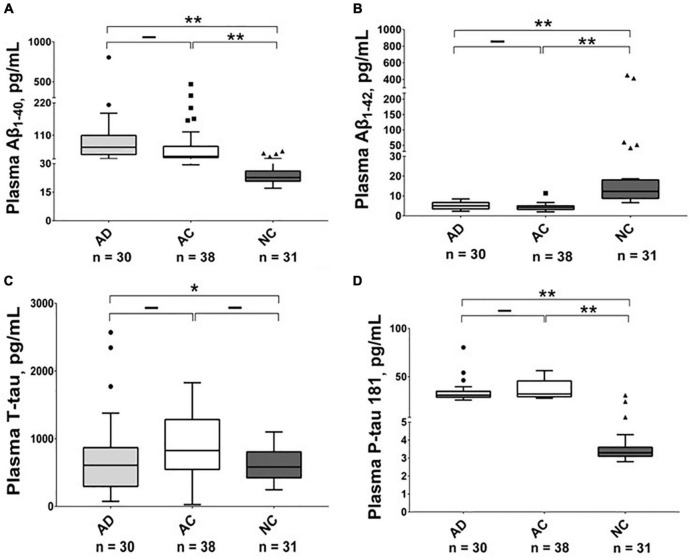
Plasma level of Aβ_1–40_, Aβ_1–42_, T-tau and P-tau 181 in different groups. The plasma concentrations of **(A)** Aβ_1–40_, **(B)** Aβ_1–42_, **(C)** T-tau and **(D)** P-tau 181 in Alzheimer’s disease (AD), adult children (AC) and non-demented control (NC) groups were compared using the Kruskal–Wallis tests. Plasma Aβ_1–42_ levels were lower in the AD and AC groups compared with the NC group, while P-tau 181 levels were higher. The boxes show interquartile range, the horizontal lines are medians and the whiskers were plotted using the Tukey method. *p < 0.05, **p < 0.01.

**FIGURE 2:**
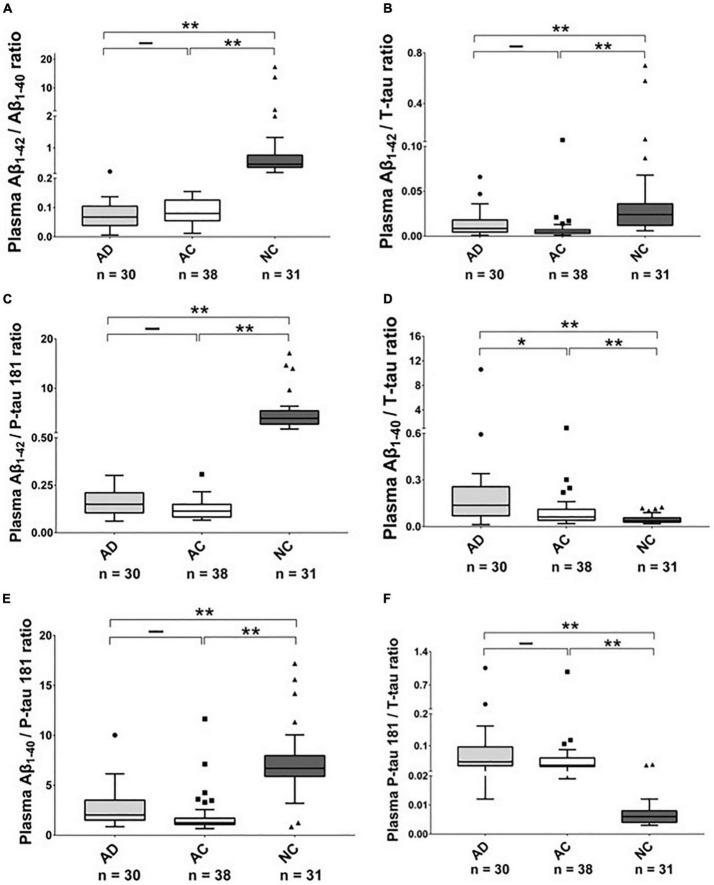
The plasma protein ratios in Alzheimer’s disease (AD), Adult Children (AC), and Non-demented Control (NC) groups. Ratio **(A)** Aβ_1–42_/Aβ_1–40_, **(B)** Aβ_1–42/_T-tau, **(C)** Aβ_1–42_/P-tau 181, **(D)** Aβ_1–40_/T-tau, **(E)** Aβ_1–40_/P-tau 181 and **(F)** P-tau 181/T-tau in these three groups were compared using the Kruskal–Wallis tests. The boxes show interquartile range, the horizontal lines are medians and the whiskers were plotted using the Tukey method. *p < 0.05, **p < 0.01.

As shown in Table 2, Figures 1, 2, and Supplementary Figures 1, 2, there were significantly higher levels of Aβ_1–40_, P-tau, and P-tau/T-tau ratio, and lower levels of Aβ_1–42_ and Aβ_1–42_/Aβ_1–40_ ratio in the AD and AC groups, compared with the NC group. No significant differences in the levels of these biomarkers were noted between the AD and AC groups, except for a higher Aβ_1–40/_T-tau ratio in the AD group (*p* = 0.019).

**TABLE 2:** Plasma biomarker levels in the studied participants.

Biomarker	AD (*n* = 30)	AC (*n* = 38)	NC (*n* = 31)	*P*-value	Pairwise comparisons
Aβ_1–40_ (pg/mL)	69.6 (45.0-109.7)	38.9 (34.5-72.5)	22.7 (20.8-26.1)	<0.001	AD = AC > NC
Aβ_1–42_ (pg/mL)	5.0 (3.5-6.7)	4.2 (3.2-5.0)	12.3 (8.8-18.1)	<0.001	AD = AC < NC
T-tau (pg/mL)	608.1 (295.8–868.2)	826.8 (547.2–1,283.7)	582.6 (423.8–805.6)	0.024	AD = AC = NC
P-tau 181 (pg/mL)	30.8 (28.7-34.9)	32.1 (29.2-45.6)	3.3 (3.1-3.6)	<0.001	AD = AC > NC
Aβ_1–42_/Aβ_1–40_	0.07 (0.04–0.1)	0.08 (0.05–0.12)	0.5 (0.4–0.7)	<0.001	AD = AC < NC
Aβ_1–42_/T-tau	0.008 (0.004–0.02)	0.005 (0.003–0.007)	0.02 (0.01–0.04)	<0.001	AD = AC < NC
Aβ_1–42_/P-tau 181	0.15 (0.1-0.2)	0.1 (0.08-0.15)	3.9 (2.7-5.4)	<0.001	AD = AC < NC
Aβ_1–40_/T-tau	0.1 (0.07–0.3)	0.06 (0.04–0.1)	0.04 (0.03–0.06)	<0.001	AD > AC > NC
Aβ_1–40_/P-tau 181	2.0 (1.5–3.5)	1.3 (1.1–1.7)	6.7 (5.9–7.9)	<0.001	AD = AC < NC
P-tau 181/T-tau	0.05 (0.03–0.09)	0.04 (0.03–0.06)	0.006 (0.004-0.008)	<0.001	AD = AC > NC

Data are shown as median (interquartile range). *P*-value for AD, AC, and NC groups using analysis of Kruskal–Wallis tests. *P* < 0.05, statistically significant, presented with higher than (>) or lower than (<).

AD, Alzheimer’s disease; AC, adult children; NC, non-demented control; Aβ, amyloid beta; T-Tau, total Tau; P-Tau181, Tau phosphorylated at threonine 181.

### The correlation of plasma Aβ_1–42_ and Aβ_1–40_ in AD and AC and between these two groups

**FIGURE 3:**
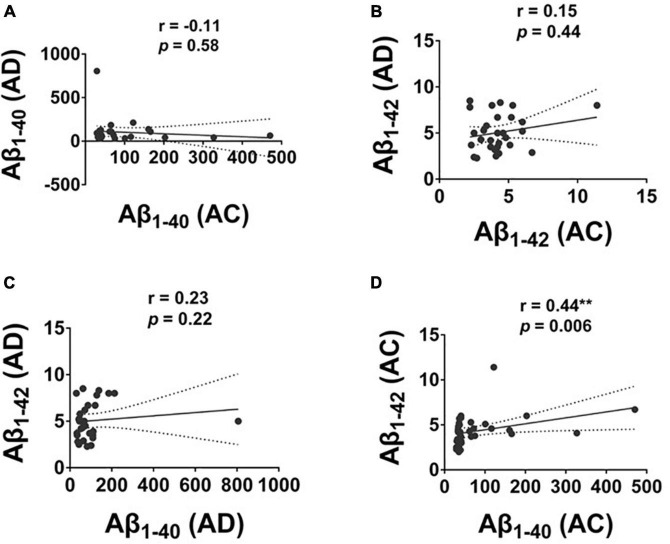
The correlation of plasma Aβ_1–42_ and Aβ_1–40_ protein levels in Alzheimer’s disease (AD) and Adult Children (AC) groups. Correlations of plasma **(A)** Aβ_1–40_, and **(B)** Aβ_1–42_ in AD and AC groups. **(C)** Aβ_1–40_ and Aβ_1–42_ in AD group and **(D)** Aβ_1–40_ and Aβ_1–42_ in AC group were assessed using the non-parametric Spearman’s rank correlation test. Graphs show regression lines with 95% confidence intervals.

The level of Aβ_1–40_ and Aβ_1–42_ was significantly correlated in the AC group with a moderate correlation (*r* = 0.44, *p* = 0.006); there was no correlation between the level of Aβ_1–40_ and Aβ_1–42_ in the AD group (Figure 3).

### The correlation of plasma T-tau and P-tau in Alzheimer’ s disease and adult children and between these two groups

**FIGURE 4:**
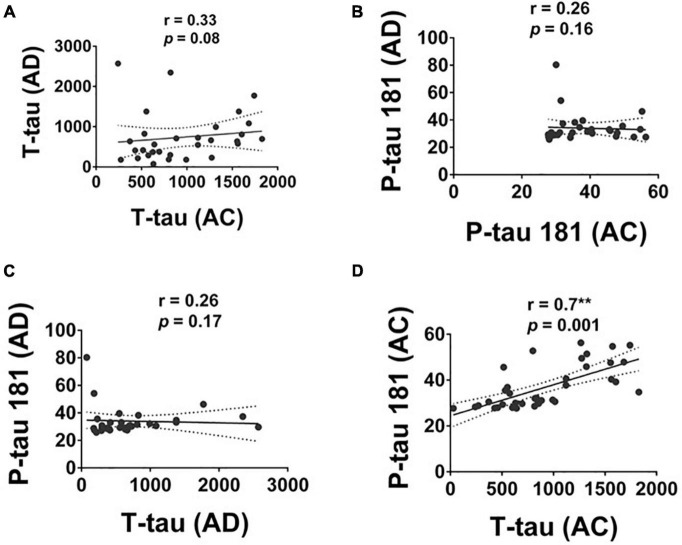
The correlation of T-tau and P-tau 181 protein levels in Alzheimer’s disease (AD), and Adult Children (AC) groups. Correlations of plasma **(A)** T-tau and **(B)** P-tau 181 in AD and AC groups. **(C)** T-tau and P-tau 181 in the AD group and **(D)** T-tau and P-tau 181 in the AC groups were assessed using the non-parametric Spearman’s rank correlation test. Graphs show regression lines with 95% confidence intervals.

The level of T-tau and P-tau were significantly correlated in the AC group with a moderate to strong correlation (*r* = 0.70, *p* = 0.001; Figure 4). No significant correlation between the level of T-tau and P-tau was observed in the AD group.

## Discussion

We have demonstrated that compared to the NC group, there was a decreased level of Aβ_1–42_ and a lower Aβ_1–42_/Aβ_1–40_ ratio, as well as an elevated level of Aβ_1–40_, and P-tau and a higher P-tau/T-tau ratio in the AD and AC groups. There was no statistical difference in these protein levels in the AD and AC groups except for a significantly higher Aβ_1–40_/T-tau ratio in the AD group compared with the AC group. The significant correlations between the levels of Aβ_1–42_ and Aβ_1–40_ and the levels of T-tau and P-tau were only in the AC group, not the AD group.

To the best of our knowledge, this is the first study to explore the plasma levels of Aβ and tau, and their concurrent correlations in AD, their AC and NC group, especially in individuals of Chinese ethnicity. Our study speculated that AC, in part, is potentially to develop AD in the view of our reports to these changes of plasma biomarkers. Our study also showed that a FH for AD was associated with a change in plasma biomarkers in cognitively normal AC, indicating their potential role as antecedent biomarkers of AD. The loss of a correlation between the level of Aβ_1–42_ and Aβ_1–40_ and the level of T-tau and P-tau could be indicative of AD clinical stage. Early diagnosis of AD even before the onset of symptoms by using these biomarkers could allow future treatments with disease-modifying effects and vigorously control modifiable cardiovascular risk factors to stop disease progression.

Unlike previous studies (Ertekin-Taner et al., 2001, 2008), a decreased plasma level of Aβ_1–42_ was noted in AC with AD parents in the current study. Ertekin-Taner et al. (2008) found that plasma Aβ_1–42_ was significantly elevated in late-onset AD first-degree relatives in comparison to unrelated controls. This discrepancy may not be related to the difference in the frequency of the APOE ε4 allele due to its similar frequency in both studies (40% in their study and 39.5% in our study), but it may be related to the age of the recruited participants. Subjects between the ages of 20 and 65 were recruited in their study, while we recruited AC aged 50–74 years old. The older age of our participants could increase the risk of amyloid deposition having started, which could present as a decline in the plasma Aβ_1–42_ and Aβ_1–42_/Aβ_1–40_ ratio (Song et al., 2011; Hendrix et al., 2021). Our results were compatible with the CSF finding in the ACS (aged 43–76 years old) conducted at Washington University, where a positive FH for AD was associated with a decrease in Aβ_1–42_ (Xiong et al., 2011). Further longitudinal studies are required to follow the trajectory of Aβ and the clinical cognitive function of cognitively intact AC.

In our study, elevated levels of Aβ_1–40_, P-tau, and P-tau/T-tau ratio were noted in the AD and AC groups compared with the NC group. These results were in line with previous studies (Ertekin-Taner et al., 2008; Janelidze et al., 2020). Increased P-tau is thought to reflect Aβ and tau pathologies in AD, while increased T-tau is a more non-specific marker of neuronal injury (Blennow et al., 2010). Therefore, P-tau and the P-tau/T-tau ratio, but not T-tau alone, may be more suitable as antecedent biomarkers of AD. Meanwhile, the fact that the AD and AC groups had similar findings, compared with the NC group, indicates the potential for AC to develop AD.

The correlation between the level of Aβ_1–42_ and Aβ_1–40_ and the level of T-tau and P-tau, only existed in AC but was lost in the AD group. Amyloid peptides are formed via amyloid precursor protein (APP) cleavage at several enzyme sites. Physiologically, the non-amyloidogenic and amyloidogenic pathways characterize the amyloid peptide formation process by APP at a dynamically steady state. The amyloidogenic pathway leads to AD. After APP cleavage by β-secretase to release β-APP, the remaining membrane-bound APP C-terminal is subsequently cleaved by γ-secretase, to release Aβ_1–40_ (90%) and Aβ_1–42_ (10%) peptide into the extracellular space (Mokhtar et al., 2013). Disruption of this steady state could lead to subsequent pathological changes in dementia. The loss of correlation between the plasma level of Aβ_1–42_ and Aβ_1–40_ in AD patients may be related to progressive Aβ_1–42_ deposition, while the correlation still exists in the preclinical stage or high potential AC. Tau protein is an integral component of the neuronal cytoskeleton and is responsible for the promotion of microtubule assembly in the normal brain. In the AD brain, tau exists in a hyperphosphorylated state, which leads to aberrant secondary structures and loss of function, resulting in a reduced ability to bind to microtubules and to promote their assembly (Mokhtar et al., 2013). Over- and hyper-phosphorylated tau may disrupt the correlation between the level of T-tau and P-tau in the AD group.

There were some limitations to the current study. First, although we have demonstrated innovative findings, they are based on a limited sample size with different demographic characteristics in each group; future studies utilizing larger samples are recommended. Most of the participants in our study were female. However, sex differences in AD plasma biomarkers during the preclinical stage is still unknown (Jack et al., 2017; Nebel et al., 2018). In addition, we did not perform a brain amyloid and Fluorodeoxyglucose (FDG) positron emission tomography (PET) scan for the AC participants to detect brain amyloid deposition and assess the presence of brain hypometabolism for confirming the role of possible pre-clinical AD; we will have to track their clinical course to determine if they are given a diagnosis of AD in the future. Besides, FDG PET may be useful to differential early onset (aged ≤65 years) and late-onset AD (aged >65 years). As compared to late-onset AD, early onset AD patients showed a significant decrease in glucose consumption in a wide portion of the left parietal lobe (Chiaravalloti et al., 2016). We should especially pay attention to those with this hypometabolism pattern for the possibility of developing AD symptoms earlier. Moreover, we did not survey all of the cardiovascular risk factors which inconclusively but might alter the result of biomarkers for AD. However, the prevalence of hypertension and diabetes are similar between AC and NC group and even higher prevalence of hypercholesterolemia and higher CAIDE dementia risk score in NC group. The possible influence of these vascular risk factors on plasma biomarkers may be minored. Family history of AD still be the major factor to influence them in our study. Finally, this was a cross-sectional examination of these biomarkers, especially focusing on whether there was a loss of correlation between the level of Aβ_1–42_ and Aβ_1–40_ and the level of T-tau and P-tau in AD. Longitudinal studies to track how these biomarkers relate to clinical presentation are encouraged.

## Conclusion

In conclusion, we reported the influence of FH for AD on a wide array of plasma biomarkers in the AC cohort of cognitively normal middle to older age individuals. Changes in the correlation between the levels of Aβ_1–42_ and Aβ_1–40_, and that of T-tau and P-tau in AC may be a biomarker for the development of AD. Further longitudinal studies following the trajectory of clinical information, plasma and CSF biomarkers, structural and functional neuroimaging, such as Aβ, tau and FDG PET scans, are required to better understand the pathogenesis of AD.

## Data availability statement

The raw data supporting the conclusions of this article will be made available by the authors, without undue reservation.

## Ethics statement

The studies involving human participants were reviewed and approved by Kaohsiung Medical University Hospital Institutional Review Board [KMUHIRB-SV(I)-20190025 and KMUHIRB-G(I)-20210026]. The patients/participants provided their written informed consent to participate in this study.

## Author contributions

T-CH and Y-HY contributed to the study conception and design. L-CH, K-YL, and Y-HY contributed to the acquisition of data. M-HC, L-CH, C-PC, and Y-HY analyzed and interpreted the data. M-HC and L-CH conducted the statistical analyses and involved in writing the initial draft of the manuscript. All authors reviewed and revised the manuscript and approved the submitted version.
