# Supplementary material for: Plasma biomarkers and their correlation in adult children of parents with Alzheimer’s disease
Source: Front Aging Neurosci. 2022 Aug 30;14:977515. doi: 10.3389/fnagi.2022.977515 (PMC9468332; doi:10.3389/fnagi.2022.977515)
Supplement: Supplementary file 1 [file Data_Sheet_1.docx]

**Supplementary Figures**


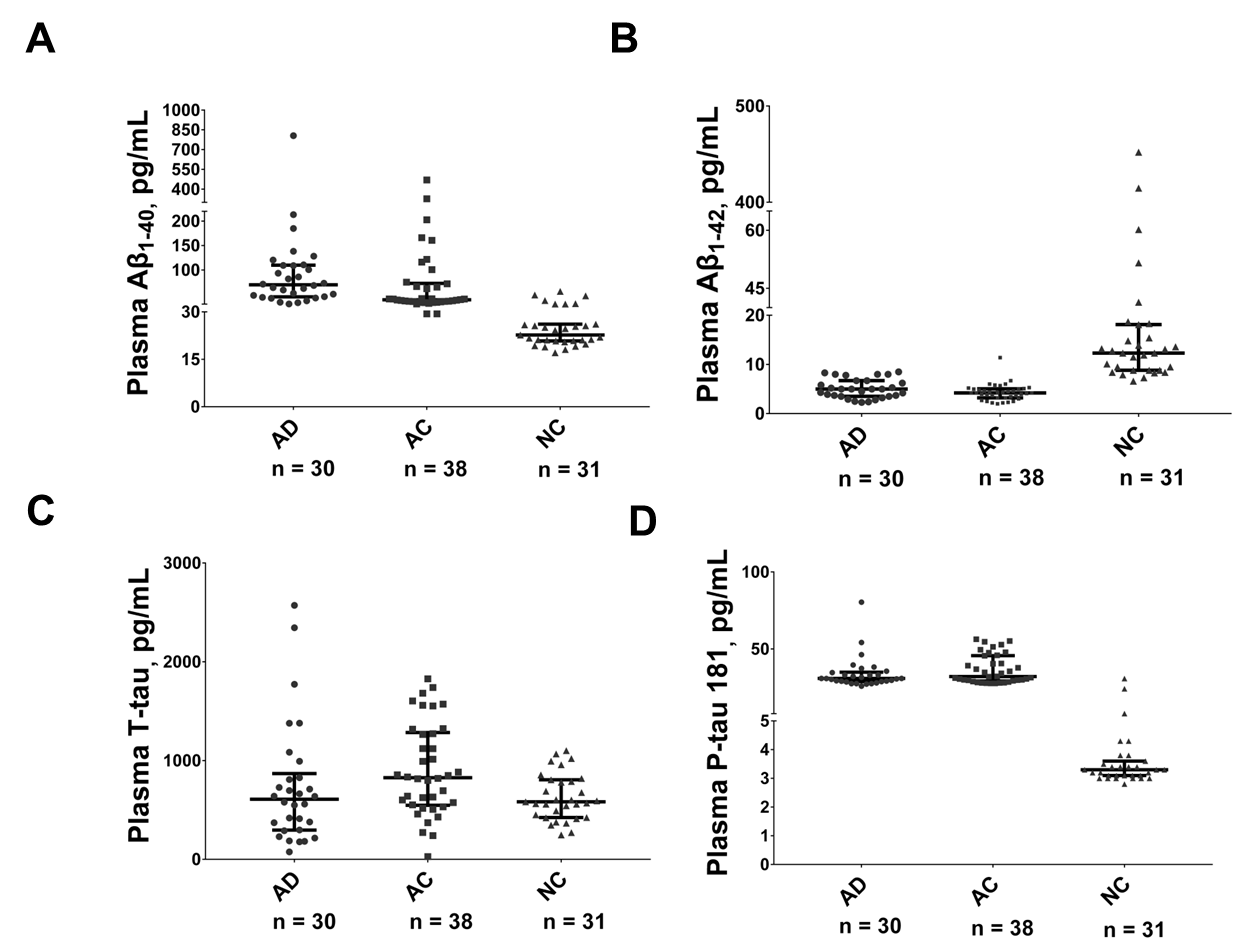


**Figure S1. Scatter plots of plasma Aβ_1-40_, Aβ_1-42_, T-tau and P-tau 181.** (A) Scatter plot for Fig. 1A. (B) Scatter plot for Fig. 1B. (C) Scatter plot for Fig. 1C. (D) Scatter plot for Fig. 1D. Solid horizontal lines represent median and error bars correspond to interquartile range in Alzheimer’s disease (AD), Adult Children (AC) and Normal Control (NC) groups.


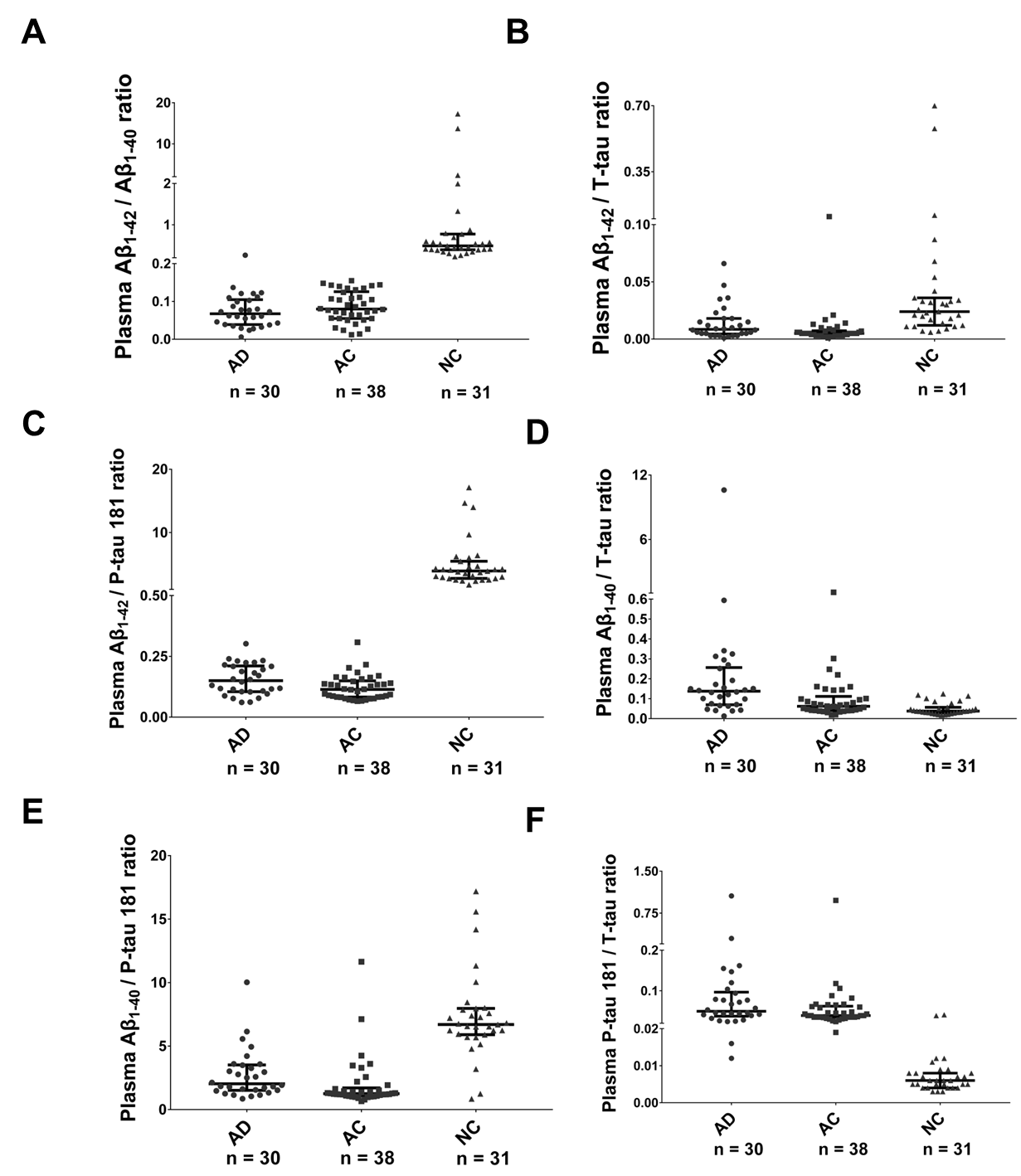


**Figure S2. Scatter plots of plasma protein level ratios Aβ_1-42_ / Aβ_1-40_, Aβ_1-42_ / T-tau, Aβ_1-42_ / P-tau 181, Aβ_1-40_ / T-tau, Aβ_1-40_ / P-tau 181 and P-tau 181 / T-tau.** A, B, C, D, E and F are scatter plots for Figs. 2A. 2B, 2C, 2D, 2E and 2F, respectively. Solid horizontal lines represent median and error bars correspond to interquartile range in Alzheimer’s disease (AD), Adult Children (AC) and Normal Control (NC) groups.
